# V1848I Mutation in the Voltage-Gated Sodium Channel Confers High-Level Resistance to Indoxacarb and Metaflumizone in *Spodoptera exigua*

**DOI:** 10.3390/insects15100777

**Published:** 2024-10-08

**Authors:** Xiangjie Liu, Minhui Cao, Wenjuan Mei, Xingliang Wang, Yidong Wu

**Affiliations:** 1 Sanya Institute of Nanjing Agricultural University, Sanya 572025, China; 2022202031@stu.njau.edu.cn (X.L.); 2023202060@stu.njau.edu.cn (M.C.); wxl@njau.edu.cn (X.W.); 2 College of Plant Protection, Nanjing Agricultural University, Nanjing 210095, China; 2021202050@stu.njau.edu.cn

**Keywords:** *Spodoptera exigua*, indoxacarb, metaflumizone, sodium channel, resistance

## Abstract

**Simple Summary:**

The role of the V1848I mutation in the voltage-gated sodium channel (VGSC) concerning SCBI resistance and inheritance patterns in *Spodoptera exigua* was investigated by developing and characterizing a near-isogenic resistant strain called WH-1848I. This mutation confers significant resistance to indoxacarb (146-fold) and metaflumizone (431-fold) in the WH-1848I strain. The SCBI resistance in this strain is autosomal, nonrecessive, and genetically linked to the V1848I mutation.

**Abstract:**

*Spodoptera exigua* is one of the most serious lepidopteran pests of global importance. With the intensive use of insecticides, *S. exigua* has evolved resistance to many insecticides, including the sodium channel blocker insecticides (SCBIs) indoxacarb and metaflumizone. In this study, we investigated the role of the V1848I mutation in the voltage-gated sodium channel (VGSC) in SCBI resistance and its inheritance patterns in *S. exigua* through the development and characterization of a near-isogenic resistant strain. The AQ-23 strain of *S. exigua*, collected in 2023 from Anqing, Anhui province of China, shows 165-fold resistance to indoxacarb compared with the susceptible WH-S strain. A frequency of 44.6% for the V1848I mutation was detected in the *SeVGSC* of the AQ-23 strain, while no F1845Y mutation was found. Through repeated backcrossing and marker-assisted selection, the V1848I mutation in the AQ-23 strain was introgressed into the susceptible WH-S strain, creating a near-isogenic strain named WH-1848I. This WH-1848I strain exhibits high levels of resistance to indoxacarb (146-fold) and metaflumizone (431-fold) but remains susceptible to broflanilide and spinosad compared with the WH-S strain. Inheritance analysis revealed that SCBI resistance in the WH-1848I strain is autosomal, nonrecessive, and genetically linked to the V1848I mutation. These findings establish a clear link between the V1848I mutation and SCBI resistance in *S. exigua*, offering valuable insights for developing molecular detection tools and resistance management strategies.

## 1. Introduction

The voltage-gated sodium channels (VGSCs) allow the entry of sodium ions into the nerve cells, leading to the occurrence of depolarization, which is crucial during the rising phase of the action potential [1,2]. The sodium channel gene was first cloned in *Electrophorus electricus* [3] and *Rattus norvegicus* [4], and the primary structures of the sodium channels were subsequently characterized. Sodium channels are composed of α subunits and β subunits. The α subunits consist of four highly homologous domains (domains I–IV), each containing six transmembrane helical segments (S1–S6), with a loop between S5 and S6 (P-loop) [2,5]. In insects, the full-length insect sodium channel gene was initially cloned from *Drosophila melanogaster* [6]. Later, sodium channel genes were cloned in many insects, such as *Musca domestica* [7], *Blattella germanica* [8] and *Bombyx mori* [9]. The structures of sodium channels have also been elucidated in the prokaryote *Arcobacter butzleri* [10] and the eukaryotes *Periplaneta americana* [11] and *E. electricus* [12], which significantly advances our understanding of the sodium channel.

Sodium channels are targets of a wide variety of both naturally occurring neurotoxins and synthetic insecticides. Insecticides developed to target sodium channels are extensively used to control agricultural, household, or medically important pests, including DDT, pyrethroids, and sodium channel blocker insecticides (SCBIs) [13]. DDT and pyrethroid enhance activation and inhibit deactivation and inactivation, resulting in prolonged channel opening [14]. SCBIs act as blockers of sodium channels to inhibit sodium channel current, with a distinct mode of action compared to DDT and pyrethroids [14].

Indoxacarb is the first commercial sodium channel blocker insecticide (SCBI) that is bioactivated by esterase or amidase into *N*-decarbomethoxyllated metabolite (DCJW) in insects [15,16,17]. DCJW blocks the generation of action potentials, ultimately causing tremors, cessation of feeding, and death in insects. Metaflumizone is a semicarbazone insecticide that also blocks VGSCs. Unlike indoxacarb, it directly blocks sodium channels by binding selectively to the slow-inactivated state [18]. SCBIs are widely used for Lepidoptera, Hemiptera, and Coleoptera pest control in the field [16,17]. However, due to their intensive use, resistance has been reported in various crop pests. To date, the APRD database shows that 19 species are resistant to indoxacarb and 7 species are resistant to metaflumizone among economically important pests, such as *Spodoptera frugiperda*, *Plutella xylostella*, *Helicoverpa armigera*, and others (Arthropod Pesticide Resistance Database, http://www.pesticideresistance.org/, accessed on 1 October 2024).

Over the past three decades, multiple point mutations in VGSCs have been reported as conferring resistance to DDT and pyrethroids in a wide range of arthropod pests [13,14]. More recently, the F1845Y and V1848I mutations in the sixth segment of domain IV of VGSC have been identified to be related to indoxacarb and metaflumizone resistance in *P. xylostella* [17,19]. Subsequently, mutations homologous to F1845Y and V1848I of *PxVGSC* were found to be associated with indoxacarb resistance in *Tuta absoluta* populations [20]. Only the V1848I mutation was found in *Liriomyza trifolii* populations, with resistance to SCBIs [21].

*Spodoptera exigua* is a worldwide polyphagous pest that is heavily reliant on chemical insecticides for its control. However, due to the extensive use of SCBIs, at least 54 cases of field-evolved resistance to indoxacarb have been reported in field populations of *S. exigua* from China and Pakistan (Arthropod Pesticide Resistance Database, http://www.pesticideresistance.org/, accessed on 1 October 2024), but the resistance mechanism remains largely elusive. Recently, the V1848I mutation, homologous to *PxVGSC* V1848I, was detected in association with indoxacarb resistance in a field population of *S. exigua* collected from Chongming Island, Shanghai, China, in 2023 [22]. In the present study, the V1848I mutation of *SeVGSC* was also identified in a field population (AQ-23) of *S. exigua* collected from Anqing, Anhui province, China, in 2023. To further investigate its role in SCBI resistance, the V1848I mutation was introgressed into the susceptible WH-S strain to establish a near-isogenic resistant strain (WH-1848I). The contribution and mode of inheritance of the V1848I mutation to resistance against the two SCBIs, indoxacarb and metaflumizone, were investigated. Our results are expected to provide a critical basis for understanding SCBI resistance mechanisms and guiding field resistance monitoring and resistance management in *S. exigua*.

## 2. Materials and Methods

### 2.1. Insects

The susceptible *S. exigua* strain (WH-S) was obtained from the Wuhan Institute of Vegetables (Wuhan, China). The WH-S strain has been maintained in the laboratory without exposure to any insecticides since it was initially collected in 1998. The AQ-23 strain of *S. exigua* was established from about two hundred late instar larvae collected from cabbage in Anqing (30.13° N, 116.71° E), Anhui province, China, in 2023.

Larvae from all populations were reared on an artificial diet with soybean flour and wheat germ as the main ingredients in the insectary at 26 ± 1 °C, a relative humidity of 60 ± 5%, and a photoperiod of 16 h light to 8 h dark. Adults were provided with a 10% (*w*/*v*) honey solution. Egg masses laid on papers were collected daily.

### 2.2. Insecticides and Bioassays

Indoxacarb (100 g/L emulsion concentrate, Jingbo Agrochemicals Technology, Binzhou, China), metaflumizone (220 g/L suspension concentrate, BASF Corporation, Shanghai, China), broflanilide (100 g/L suspension concentrate, BASF Corporation, Shanghai, China), and spinosad (25 g/L suspension concentrate, Corteva Agrosciences, Shanghai, China) were used for bioassays.

Specific details of biological assays can be found in Zuo et al. (2021) [23] and Mei et al. (2023) [24]. Each insecticide was diluted into five to seven concentrations using distilled water containing 0.1% (*w*/*v*) Triton X-100. A control treatment was prepared with distilled water containing 0.1% Triton X-100. The heated artificial diet was poured into a 24-well plate (with a surface area per well of ∼2 cm^2^; Merck Life Science, Shanghai, China), and then 100 μL of insecticide solution or 0.1% Triton X-100 solution was added to the surface of the cooled diet and left at room temperature to dry. One third-instar larva was placed in each well, with a total of 48 larvae tested for each concentration. The plates were maintained under the same conditions as the rearing environment. After treatment for 72 h, mortality was assessed. If a larva did not move after being gently probed with a brush, it was recorded as dead. If the 95% fiducial limits did not overlap for LC_50_ values from a probit analysis performed using SPSS software (version 25.0; IBM Corp., Armonk, NY, USA), the susceptibility of the populations was considered significantly different [25].

### 2.3. Detection of the Resistance Allele of SeVGSC

**Figure 1:**
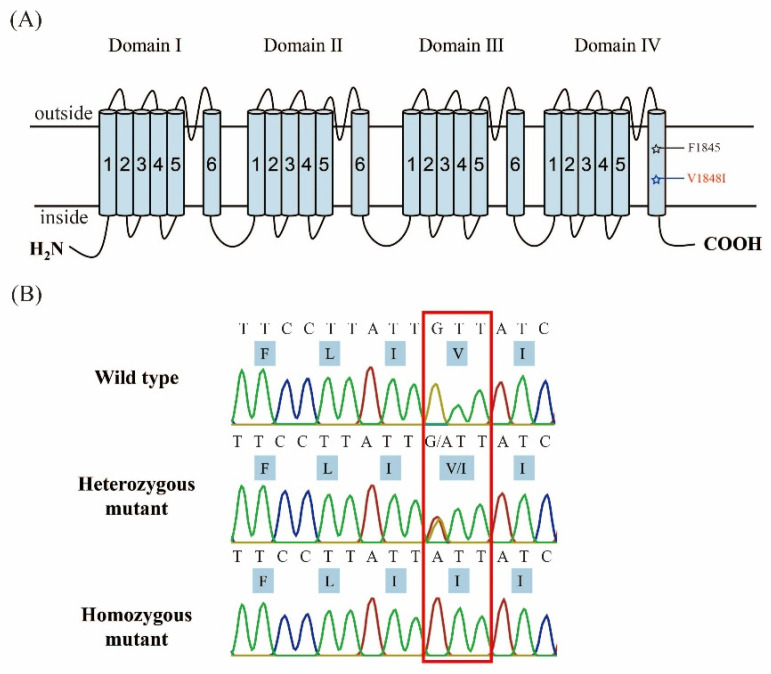
Structure of *SeVGSC* and partial sequencing chromatograms with F1845 and V1848 sites. (**A**) The sodium channel consists of four main domains, each containing six transmembrane segments. The two mutations related to SCBI insecticide resistance are indicated by pentagrams. The blue pentagram represents detected mutations, and the black pentagram represents undetected mutations. Positions are numbered according to the amino acid sequence of the sodium channel protein from *P. xylostella* (GenBank accession no. KM027335) following the reference of Wang et al. [17]. (**B**) Representative chromatograms of direct sequencing of the polymerase chain reaction products for genotyping the V1848I mutation of *SeVGSC*. The position of the V1848I mutation is boxed. Two peaks (representing GTT and ATT) indicate that these samples contained a mix of V1848 and I1848 alleles.

The genomic DNA of the larvae or adult hindlegs of *S. exigua* was extracted using DNAiso Reagent kits (Takara, Otsu, Japan). The mutations of F1845Y and V1848I (named according to the VGSC of *P. xylostella*) were identified by direct sequencing of the PCR products, which were amplified with a pair of specific primers (VGSC-F: 5′-ATGTCAACATCAGCCGGA-3′; VGSC-R: 5′-CTGTGAGTAATTCTCGAGAATG-3′). The PCR reaction solution consisted of 12.5 μL of 2× Taq Master Mix (Vazyme, Nanjing, China), 1 μL of each primer (10 μmol/L), 6 μL of template genomic DNA, and 4.5 μL of ddH_2_O in a final volume of 25 μL. The PCR amplification was performed with the following procedure: 95 °C for 3 min; 34 cycles of 95 °C for 20 s, 54 °C for 20 s, and 72 °C for 10 s; and 72 °C for 5 min. The PCR products were then directly sequenced with the forward primer by Tsingke Biological Technology (Sanya, China). The genotypes of *SeVGSC* were determined according to their sequence chromatograms (Figure 1B).

### 2.4. Genetic and Linkage Analysis of Resistance to SCBIs

Establishing a near-isogenic WH-1848I strain, the female and male pupae of the WH-S strain and WH-1848I strain were separated for reciprocal crosses. The virgin female adults of the WH-1848I strain were mass-crossed with the male adults of the WH-S strain and vice versa (30 pairs each). The degree of dominance (*D*) was calculated using the following formula [26]: *D* = (2*X*_2_ − *X*_1_ − *X*_3_)/(*X*_1_ − *X_3_*), where *X*_1_, *X*_2_, and *X*_3_ represent the log (LC_50_) values of the resistant strain, F_1_ hybrids, and the susceptible strain, respectively. The *D* values ranged from −1 (completely recessive) to +1 (completely dominant).

The male adults of the F_1_ (from WH-1848I males crossed with WH-S females) were mass-crossed with virgin female adults of WH-S to produce BC progeny. Subsequently, a total of 240 third-instar larvae from the BC progeny were treated with 2 mg/L and 4 mg/L of indoxacarb and metaflumizone, respectively, to investigate the association between the V1848I mutation of *SeVGSC* and SCBI resistance. After 72 h of treatment, 30 larvae were randomly collected from both the survivors and the untreated group for genotyping at site 1848 of *SeVGSC*.

## 3. Results

### 3.1. The Indoxacarb Resistance Level of AQ-23 Strain 

Resistance to indoxacarb in the F_1_ progeny of the AQ-23 strain of *S. exigua* is 165-fold compared with the susceptible WH-S strain (Table 1).

### 3.2. Identification and Frequency of the SeVGSC V1848I Mutation in the AQ-23 Strain

The 1845F and 1848V alleles are located in the sixth transmembrane segment of the domain Ⅳ of the wild-type *SeVGSC* (Figure 1A). The F1845Y and V1848I mutations in the AQ-23 strain were screened through direct sequencing of the PCR products flanking the mutation sites. Among 28 F_1_ larvae genotyped, 7 individuals were wild-type homozygotes for 1848^V/V^ (GTT), 17 individuals were heterozygous for the mutation 1848^V/I^ (GTT/ATT), and 4 individuals were homozygous for the mutation 1848^I/I^ (ATT). The mutation frequency of the *SeVGSC* 1848I allele was 44.6% in the F_1_ of AQ-23, but no F1845Y mutation was detected.

### 3.3. Introgression of the 1848I Allele into the Susceptible WH-S Strain

**Figure 2:**
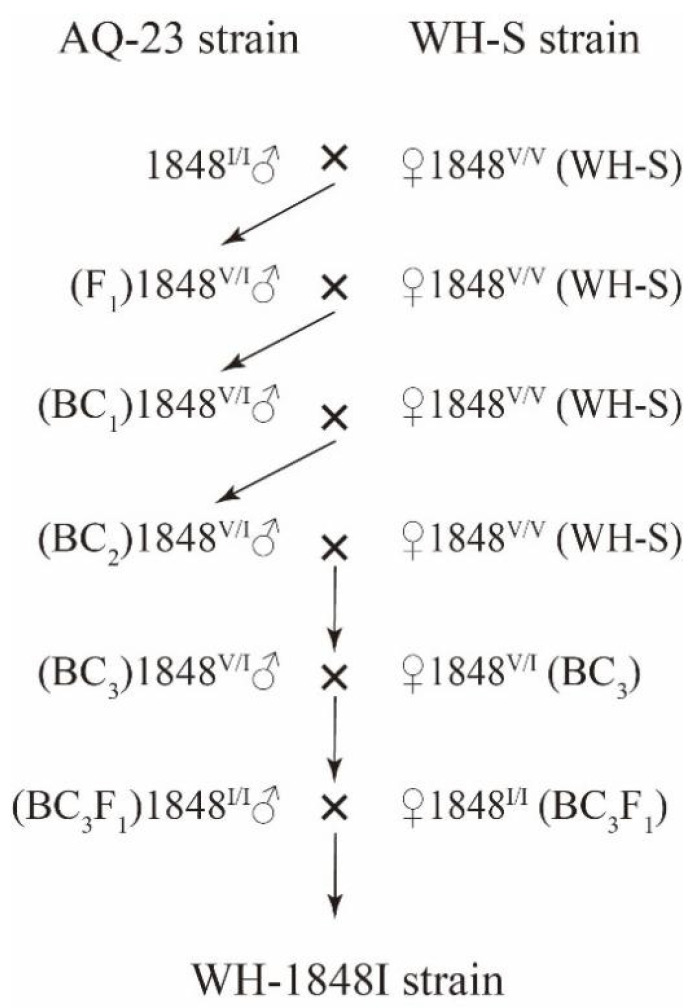
The crossing design for introgression of the V1848I mutant allele of *SeVGSC* from the resistant AQ-23 population into the susceptible WH-S strain of *Spodoptera exigua*.

In order to eliminate the interference of other resistance genes, a near-isogenic WH-1848I strain was established through repeated backcrossing with WH-S. The marker-assisted introgression strategy is illustrated in Figure 2. The moths used for the crossing schemes were genotyped nondestructively before mating by removing one hind leg for PCR and sequence analysis. 

A total of 10 male adults carrying the homozygous 1848^I/I^ mutation isolated from 123 adults of the AQ-23 strain were mass-mated with 10 virgin female adults from the WH-S strain (homozygous for the wild type 1848^V/V^) to generate F_1_ progeny (1848^V/I^). Subsequently, 30 heterozygous F_1_ male adults (1848^V/I^) were backcrossed with 30 virgin female adults of WH-S to generate BC_1_ progeny, and 30 BC_1_ males, which were heterozygous for the mutation (1848^V/I^), were backcrossed with 30 virgin females from WH-S to create BC_2_ progeny. To create BC_3_ progeny, 30 male heterozygotes of BC_2_ (1848^V/I^) were backcrossed with 30 virgin females of WH-S. After three generations of backcrossing, the male heterozygous individuals (1848^V/I^) of BC_3_ were mass-mated with female heterozygous individuals (1848^V/I^) of BC_3_ to produce the BC_3_F_1_. The sib-mating of homozygous BC_3_F_1_ (1848^I/I^) generated a near-isogenic strain named WH-1848I, sharing 94% of the genetic background with WH-S.

### 3.4. Inheritance of SCBI Resistance in the Near-Isogenic WH-1848I Strain

Compared with the susceptible WH-S strain, the resistance ratios of WH-1848I for indoxacarb and metaflumizone were 146-fold and 431-fold, respectively. The LC_50_ values to indoxacarb (5.522 and 6.139 mg/L) and metaflumizone (14.815 and 18.298 mg/L) were not significantly different between the hybrid F_1a_ and F_1b_, respectively, as indicated by their overlapped LC_50_’s 95% fiducial limits. The dominance value was 0.33 for indoxacarb resistance, and −0.03 for metaflumizone (Table 2). These results indicated that the SCBI resistance is autosomal and nonrecessive (incompletely dominant to indoxacarb, semi-dominant to metaflumizone) in the WH-1848I strain of *S. exigua*.

### 3.5. Genetic Linkage between the V1848I Mutation of SeVGSC and SCBI Resistance 

**Figure 3:**
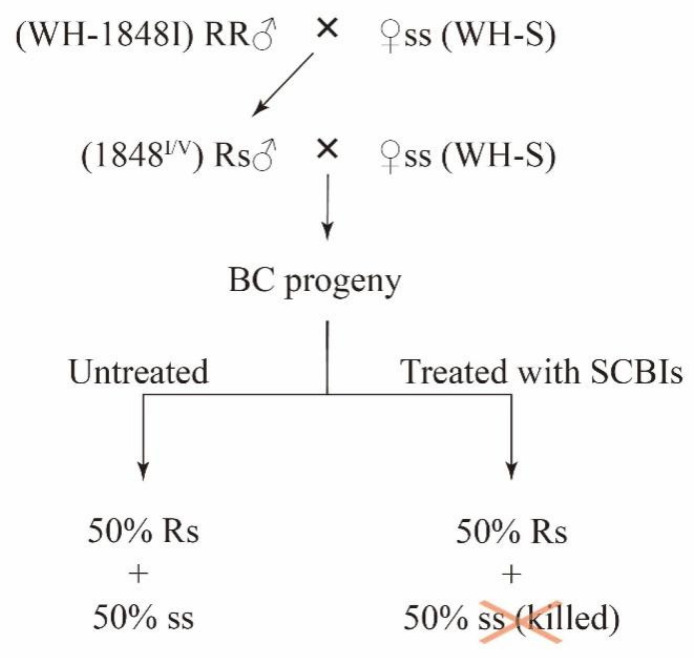
Diagram of genetic linkage of SCBIs resistance and V1848I mutation of *SeVGSC*. The BC progeny was divided into two groups: untreated and treated with SCBIs.

Genetic linkage analysis was performed in the near-isogenic WH-1848I strain to determine whether the V1848I mutation is linked to SCBI resistance (Figure 3). The survival rates of BC progeny treated with 2 and 4 mg/L indoxacarb were 46.7% (112/240) and 31.7% (76/240), respectively. Thirty randomly selected survivors all exhibited a heterozygotic genotype for 1848^V/I^. Similarly, the survival rates of BC progeny treated with 2 and 4 mg/L metaflumizone were 53.3% (128/240) and 40.4% (97/240), respectively, with 30 randomly selected survivors having a heterozygotic genotype for 1848^V/I^ (Table 3). These results confirmed that the SCBI resistance is tightly linked to the V1848I mutation of *SeVGSC* in the near-isogenic WH-1848I strain.

### 3.6. No Cross-Resistance to Broflanilide and Spinosad in the WH-1848I Strain

The WH-1848I strain had high levels of resistance to indoxacarb (146-fold) and metaflumizone (431-fold) compared with the susceptible WH-S strain, but it was still susceptible to broflanilide (1.4-fold) and spinosad (1.5-fold) (Table 4).

## 4. Discussion

Sodium channel mutations in insects are linked to resistance to pyrethroids and SCBIs. The Leu to Phe (L1014F) mutation in the S6 of domain II and the Met to Thr (M918T) mutation in the S4–S5 loop of domain II of the sodium channel have been associated with *kdr* resistance to pyrethroids in numerous insects, such as *Blattella germanica* [27], *M. domestica* [7], and *P. xylostella* [28,29]. The F1845Y and/or V1848I mutations, associated with SCBI resistance, are located in the S6 of domain IV of the sodium channel in *P. xylostella* [17,19], *T. absoluta* [20], *L. trifolii* [21], and *S. exigua* [22]. These findings suggest that both pyrethroids and SCBIs act on the sodium channel, but at different sites of action [13,30,31], indicating no cross-resistance between pyrethroids and SCBIs.

In order to eliminate the potential influence of other minor resistance factors and genetic backgrounds on the target resistance mutations, the construction of near-isogenic strains or CRISPR-based knock-in strains is typically used to assess the role and inheritance mode of resistance mutations. Wang et al. [32] employed CRISPR/Cas9 technology to introduce the I4790M mutation into the ryanodine receptor of a susceptible strain of *P. xylostella* and found that the knock-in strain obtained 40.5-fold resistance to flubendiamide, with a dominance value of around −0.8. Similarly, Jiang et al. [33] constructed a near-isogenic I4790M strain of *P. xylostella*, which displayed 57-fold resistance to flubendiamide with a dominance value of around −0.5. These studies indicate that both gene introgression and the knock-in approach are effective for determining the contribution of resistance mutations. Zhang et al. [22] isolated the SH23-S2 strain of *S. exigua* by laboratory selection of a field-collected population. The SH23-S2 strain is homozygous for the V1848I mutation and exhibits 876-fold resistance to indoxacarb compared with the WH-S strain. In the present study, the WH-1848I strain shows 146-fold resistance to indoxacarb compared to the WH-S strain. The WH-1848I strain of *S. exigua* was constructed using the gene introgression approach, achieving 94% genetic background similarity to the susceptible WH-S strain, which largely minimizes the influence of other resistance factors and genetic background. Therefore, the 146-fold resistance to indoxacarb could more accurately be attributed to the V1848I mutation in *S. exigua*. Additionally, our study revealed that the V1848I mutation confers a higher level of resistance (431-fold) to metaflumizone.

Target-site mutations often result in recessive resistance, such as those in the ryanodine receptor [24,32,33,34,35] and the nicotinic acetylcholine receptor α6 subunit [36,37,38]. A previous study in *P. xylostella* showed that indoxacarb resistance conferred by V1848I mutation was codominant, whereas metaflumizone resistance was incompletely recessive [19]. Differences in the degree of dominance of V1848I-mediated resistance to indoxacarb and metaflumizone were also observed in this study, with incomplete dominance against indoxacarb and codominance against metaflumizone in the WH-1848I strain of *S. exigua*. The variation in the dominance of indoxacarb resistance between the two pests may be due to species differences or any other unknown reasons, which deserves further investigation.

In addition to target-site mechanisms, metabolic mechanisms associated with SCBI resistance have been reported in several species, such as *P. xylostella* [39], *S. exigua* [40], *Choristoneura rosaceana* [41], *S. litura* [42], and *H. armigera* [43]. In this study, the indoxacarb resistance of the field-collected AQ-23 strain and the near-isogenic WH-1848I strain of *S. exigua* were 165-fold and 146-fold, respectively. The frequency of the 1848I mutation was 100% in the WH-1848I strain but 44.6% in the AQ-23 strain, indicating the involvement of potential metabolic resistance in the AQ-23 strain. However, the molecular mechanism of metabolic resistance to SCBIs is largely unknown, and further studies are needed in the future.

## 5. Conclusions

A near-isogenic WH-1848I strain of *S. exigua* was constructed using a gene introgression approach, which precisely revealed the relationship between the V1848I mutation of *SeVGSC* and SCBI resistance. Genetic analyses demonstrated that V1848I is tightly linked to SCBI resistance, conferring incompletely dominant resistance to indoxacarb and co-dominant resistance to metaflumizone. Broflanilide and spinosad can be utilized to control resistant populations caused by V1848I mutation in *S. exigua* because there is no cross-resistance between these two insecticides and SCBIs. Our results will not only enhance the understanding of SCBI resistance mechanisms but also help develop molecular monitoring methods and resistance management strategies for *S. exigua*.

## Figures and Tables

**Table 1:** Toxicity of indoxacarb against the susceptible WH-S strain and the resistant AQ-23 strain of *Spodoptera exigua*.

Strain	N	Slope ± SE ^a^	LC_50_ (95%FL) (mg/L) ^b^	RR ^c^
WH-S	288	5.60 ± 0.60	0.213 (0.186–0.243)	
AQ-23	288	1.74 ± 0.20	35.036 (27.645–46.811)	165

^a^ Standard error. ^b^ LC_50_, median lethal concentrations; FL, fiducial limit. ^c^ Resistance ratio = LC_50_ of AQ-23/LC_50_ of WH-S.

**Table 2:** Toxicity of indoxacarb and metaflumizone against the susceptible WH-S strain, the near-isogenic WH-1848I strain, and the F_1_ progeny from reciprocal crosses of *Spodoptera exigua*.

Strain/Cross	N	Slope ± SE ^a^	LC_50_ (95%FL) (mg/L) ^b^	RR ^c^	*D* ^d^
**Toxicity of indoxacarb**					
WH-S (S)	288	5.60 ± 0.60	0.213 (0.186–0.243)		
WH-1848I (R)	288	2.72 ± 0.29	31.174 (20.490–46.453)	146	
F_1a_ (R♂ × S♀)	288	3.52 ± 0.38	5.522 (4.771–6.394)	26	0.31
F_1b_ (R♀ × S♂)	288	3.07 ± 0.34	6.139 (5.246–7.227)	29	0.35
Pooled F_1_	576	3.27 ± 0.25	5.819 (4.399–7.775)	27	0.33
**Toxicity of metaflumizone**					
WH-S (S)	288	3.76 ± 0.43	0.881 (0.765–1.020)		
WH-1848I (R)	288	2.22 ± 0.25	379.278 (307.929–460.023)	431	
F_1a_ (R♂ × S♀)	288	1.97 ± 0.25	14.815 (11.914–19.278)	17	−0.07
F_1b_ (R♀ × S♂)	288	2.27 ± 0.29	18.298 (14.933–23.587)	21	0.00
Pooled F_1_	576	2.08 ± 0.19	16.545 (14.198–19.750)	19	−0.03

^a^ Standard error. ^b^ LC_50_, median lethal concentrations; FL, fiducial limit. ^c^ Resistance ratio = LC_50_ of WH-1848I or F_1_/LC_50_ of WH-S. ^d^ Degree of dominance, ranges from −1 (completely recessive) to +1 (completely dominant).

**Table 3:** Genetic linkage analysis of the V1848I mutation of *SeVGSC* and SCBI resistance in the near-isogenic WH-1848I strain of *Spodoptera exigua*.

Treatment	Survival (%)	Number of Larvae Genotyped	Number of Larvae for Each Genotype	
			1848^V/I^ (Mutant)	1848^V/V^ (Wild Type)
Indoxacarb				
Untreated group		30	18	12
Survivors at 2 mg/L	46.7 (112/240)	30	30	0
Survivors at 4 mg/L	31.7 (76/240)	30	30	0
Metaflumizone				
Untreated group		30	18	12
Survivors at 2 mg/L	53.3 (128/240)	30	30	0
Survivors at 4 mg/L	40.4 (97/240)	30	30	0

**Table 4:** Toxicity of broflanilide and spinosad against the susceptible WH-S strain and the near-isogenic WH-1848I strain of *Spodoptera exigua*.

Strain	Insecticide	N	Slope ± SE ^a^	LC_50_ (95%FL) (mg/L) ^b^	RR ^c^
WH-S	Broflanilide	288	4.53 ± 0.53	0.034 (0.030–0.039)	
	Spinosad	288	2.95 ± 0.28	0.734 (0.546–0.990)	
WH-1848I	Broflanilide	240	3.32 ± 0.35	0.047 (0.041–0.055)	1.4
	Spinosad	240	2.51 ± 0.27	1.079 (0.903–1.292)	1.5

^a^ Standard error. ^b^ LC_50_, median lethal concentrations; FL, fiducial limit. ^c^ Resistance ratio = LC_50_ of WH-1848I/LC_50_ of WH-S.

## Data Availability

The original contributions presented in the study are included in the article; further inquiries can be directed to the corresponding authors.
